# A novel copro-diagnostic molecular method for qualitative detection and identification of parasitic nematodes in amphibians and reptiles

**DOI:** 10.1371/journal.pone.0185151

**Published:** 2017-09-21

**Authors:** Lucas G. Huggins, Christopher J. Michaels, Sheena M. Cruickshank, Richard F. Preziosi, Kathryn J. Else

**Affiliations:** 1 Faculty of Biology, Medicine and Health, University of Manchester, MAHSC, Manchester, United Kingdom; 2 Herpetology Section, ZSL London Zoo, London, United Kingdom; 3 Faculty of Science and Engineering, Manchester Metropolitan University, Manchester, United Kingdom; Universidade de Aveiro, PORTUGAL

## Abstract

Anthropogenic disturbance via resource acquisition, habitat fragmentation and climate change, amongst other factors, has led to catastrophic global biodiversity losses and species extinctions at an accelerating rate. Amphibians are currently one of the worst affected classes with at least a third of species categorised as being threatened with extinction. At the same time, they are also critically important for many habitats and provide man with a powerful proxy for ecosystem health by acting as a bioindicator group. Whilst the causes of synchronised amphibian losses are varied recent research has begun to highlight a growing role that macroparasites are playing in amphibian declines. However, diagnosing parasite infection in the field can be problematic, principally relying on collection and euthanasia of hosts, followed by necropsy and morphological identification of parasites *in situ*. The current study developed a non-invasive PCR-based methodology for sensitive detection and identification of parasitic nematode DNA released in the faeces of infected amphibians as egg or tissue fragments (environmental DNA). A DNA extraction protocol optimised for liberation of DNA from resilient parasite eggs was developed alongside the design of a novel, nematode universal, degenerate primer pair, thus avoiding the difficulties of using species specific primers in situations where common parasite species are unknown. Used in conjunction this protocol and primer pair was tested on a wide range of faecal samples from captive and wild amphibians. The primers and protocol were validated and detected infections, including a *Railletnema* nematode infection in poison dart frogs from ZSL London Zoo and *Mantella cowani* frogs in the wild. Furthermore, we demonstrate the efficacy of our PCR-based protocol for detecting nematode infection in other hosts, such as the presence of pinworm (*Aspiculuris*) in two tortoise species and whipworm (*Trichuris muris*) in mice. Our environmental DNA approach mitigates problems associated with microscopic identification and can be applied to detect nematode parasitoses in wild and captive hosts for infection surveillance and maintenance of healthy populations.

## Introduction

Worldwide, there is increasing scientific recognition of dramatically elevated extinction rates in modern species and a growing biodiversity crisis [1–3]. Butchart et al. (2010) comprehensively reviewed global indicators of biodiversity trends, finding that 80% of state indicators exhibited negative trends towards reduced biodiversity and that species extinction risk was actually accelerating. Of all animal classes, amphibians best exemplify the current biodiversity crisis as a third of extant species are categorised as being threatened by extinction by the IUCN with many more as yet Data Deficient [3,4]. The causes of declines in amphibians, alike to declines in other classes, are multifactorial principally originating from anthropogenic ecosystem alteration via habitat alteration or degradation, climate change, pollution and introduction of alien species and novel diseases [3,5–7]. Now, more research has focused on a growing understanding of the importance of macroparasite infections that contribute alongside anthropogenic factors to cause amphibian extirpations and extinctions [8–11]. For example, the trematode *Ribeiroia ondatrae*, is now recognised as the principal causative agent for widespread outbreaks of severe limb deformities in many different North American frog populations, causing high levels of mortality [12,13]. Other culprits include members of the trematode genera *Echinostoma* and *Echinoparyphium* that are found in wetland habitats worldwide, infecting a range of anuran hosts. These species cause stunted growth and oedema in tadpoles, renal pathology in adult frogs and have been observed to reach infection prevalence as high as 100% in some zones [14]. Furthermore, captive amphibian populations have been reported to die-off after succumbing to *Rhabdias bufonis* or *R*. *tokyoensis* lungworm infection [15,16]. The opportunistic spread of a native or newly introduced macroparasite can be the final insult to an already weakened amphibian community that has been previously damaged by more pervasive pathogens, for example *R*. *ondatrae* acting in synchrony with the widespread fungal pathogen *Batrachochytrium dendrobatidis* (Bd) [10].

Given the importance of amphibian parasites in species decline and ecological dynamics, it is surprising that they are relatively under researched [9]. Research attempts have primarily been hampered by difficulties in identification, which is traditionally done based on morphology [17]. Morphological identification requires high levels of expertise and is very susceptible to human error, due to interspecific similarity in egg and larval stage morphology [17,18]. To overcome this, PCR-based diagnostics can be used which are more sensitive and less time-consuming than microscopy [19–21].

Parasitological studies today are now beginning to focus more on non-invasive sampling, involving collection of “environmental DNA or eDNA” that is shed and left behind by the host under investigation; faeces is a particularly rich source due to the frequent presence of excreted parasite transmissible stages [8,22]. Copro-diagnosis, the analysis of faeces for parasite life cycle stages and eDNA, is a particularly attractive non-invasive technique as samples can easily be collected *in situ* and species diagnostic eDNA can be targeted which also identifies the infective species i.e. DNA-barcoding [19,23–25].

However, amphibian host-parasite systems are poorly characterised making the use of broad-spectrum primers crucial that target higher taxonomic ranks instead of species specific ones [17,26,27]. We report here the development of a novel pair of DNA-barcoding primers suitable for selective amplification of nematode DNA from across the Amphibia class and used in the context of a copro-diagnostic protocol. Furthermore, we highlight the efficacy of this copro-diagnostic protocol in identification of parasites from other host-parasite systems, such as reptiles and mammals, with potential applications as a conservation or veterinary tool in these groups as well.

## Materials and methods

Mouse models of *Trichuris muris* and *Trichinella spiralis* nematode infection were initially used to develop an effective DNA extraction and detection protocol and also used to test designed primer specificity. *T*. *spiralis* was maintained at the University of Manchester as described previously [28]. The Edinburgh isolate of *T*. *muris* [29] was used throughout, and has been maintained at the University of Manchester since 1989. Non-infected mice provided a negative control to further ascertain protocol specificity. Once an effective protocol had been established samples from individuals of a variety of amphibian and reptile species (see below) with an unknown infection status were analysed. The protocol developed was logged in protocols.io accessible via http://dx.doi.org/10.17504/protocols.io.i32cgqe.

### Sources of faecal samples

Faeces were collected from mice experimentally infected with a dosage of 200 *T*. *muris* eggs or 200 *T*. *spiralis* infective larvae as part of other, ongoing experiments at the University of Manchester under the under the Home Office project licence 70/8127 and regulation of the Home Office Scientific Procedures Act (1986). Faeces were also collected from known non-infected mice, to act as negative controls. All animal experiments were approved by the University of Manchester Animal Welfare and Ethical Review Board.

Faecal samples from amphibian and reptile hosts with an unknown infection status were collected for analysis from several sources. Twelve *Mantella betsileo* frogs purchased from the pet trade in November 2015, two months after capture from the wild, were maintained and kept separate from other species colonies by one of the authors (RP) at the University of Manchester. Faecal samples were collected weekly from these individuals to allow for optimisation of conditions for the copro-diagnostic protocol’s DNA extraction steps. In addition, faecal samples from wild *Mantella cowani* individuals were collected in December 2015 from fieldwork in Madagascar under the research permit 309/15/MEEF/SG/DGF/DCD.SAP/SCB (granted 20th of November 2015) and kept in RNAlater (Thermofisher, Loughborough, UK) for three weeks until shipping to the UK.

Samples from 24 amphibian and reptile species S1 Table maintained at ZSL London Zoo were also used, following freezing and delivery to the University of Manchester for processing, two weeks post-collection.

### DNA extraction from tissue

Nematode tissue DNA was extracted to test for primer functionality in amplifying nematode DNA. DNA was extracted from 15 mg of *T*. *muris* tissue using the QIAGEN DNeasy® Blood & Tissue Kit (Manchester, UK) under aseptic conditions with only slight modifications to the manufacturer’s protocol. The DNA was allowed to elute for 15 min into 200 μl of buffer AE on the spin column membrane during the final step of the extraction protocol. When not in use DNA samples were kept chilled at 4°C.

### DNA extraction from faeces and DNA concentration analysis

DNA was extracted from a starting faecal quantity of 10–200 mg (depending on obtainable amount) using the QIAamp® Fast DNA Stool Mini Kit (Qiagen) under aseptic conditions using the manufacturer’s protocol alongside the following modifications. A disruption step was included in which the faecal samples were added to 1 ml of InhibitEx buffer followed by bead-beating using 4 mm diameter borosilicate glass beads (Sigma) placed within an Eppendorf Safelock 2 ml test tube. Samples were then bead-beaten in a Retsch MM400 mixer mill (Derbyshire, UK) at 30 Hz for between 5–10 min with regular movement of the samples between the pockets of the arm cradles to ensure a consistent beating across all samples. Next, samples were vortexed for one minute and then incubated and shaken in an Eppendorf Thermomixer C (Stevenage, UK) at 45°C and 67 g for between 1–2 hours. The Proteinase K digestion was carried out for 20 min. Two elution steps were typically carried out, a first elution for 20 min in 100 μl of buffer AE with centrifugation, followed by a second elution step in 50 μl for 15 min and centrifugation. When not in use DNA samples were kept chilled at 4°C. After the incubation and centrifugation steps the beads were removed and washed in Virkon, followed by a 10% HCl acid bath and then Milli-Q water (from Millipore Advantage A10, Feltham, UK) to allow for their re-use. DNA concentration analysis was performed on a ThermoFisher Scientific NanoDrop 2000 spectrophotometer.

### Development of nematode universal barcoding primers

A comprehensive list of common parasitic nematodes that infect wild animals, such as amphibians and reptiles, was compiled, consisting of a large range of different families and genera from the Nematoda phylum Table 1. The 18S ribosomal RNA (rRNA) gene was chosen as a target region as it is commonly used in nematode DNA barcoding studies and has proven to be more useful than the mitochondrial cytochrome oxidase 1 (COI) gene in the Nematoda phylum [30–32]. Fungal species, especially from the Basidiomycota, were also selected as these are known to have 18S rRNA sequences that commonly cross-react with primers designed to be nematode specific [19,27]. Amphibian 18S rRNA sequences were included as any designed primers must not amplify host DNA Table 1. Sequences were taken from the GenBank database and aligned in the sequence visualisation program BioEdit v7.2.5 (http://www.mbio.ncsu.edu/bioedit/page2.html) to find regions conserved within all of the nematode species but absent in the fungi and amphibian sequences. Primers were designed for the loci of the conserved regions and degenerate base pairs added to the sequences to increase the possible range of nematode 18S sequences they could target. The degenerate primer sequences were analysed using OligoAnalyzer 3.1 (www.idtdna.com/calc/analyzer) and optimised. 15 degenerate primers were designed and these were tested in 28 different combinations. Combinations were only chosen if they amplified fragments larger than 100 bp and smaller than 700 bp and had mean melting temperatures within approximately 5°C of each other.

**Table 1:** List of species used in primer design alignment.

Nematodes	Fungi
*Trichuris muris*	*Sidera vulgaris*
*Trichuris trichiura*	*Sidera lenis*
*Trichinella spiralis*	*Herpotrichiellaceae* sp.
*Paratrichosoma sp*.	*Exophiala xenobiotica*
*Dicotophyme renale*	*Exophiala castellanii*
*Eustrongylides ignotus*	*Onslowia edophytica*
*Rhabdias bufonis*	*Lulworthia fucicola*
*Rhabditis* sp.	*Corollospora maritima*
*Ascaris lumbricoides*	*Acremonium strictum*
*Ascaris suum*	*Acremonium asperulatum*
*Strongyloides stercoralis*	*Lindra obtusa*
*Strongyloides procyonis*	*Lindra marinera*
*Strongyloides ratti*	*Metarhizium anisopliae*
*Cosmocercoides dukae*	*Aspergillus niger*
*Parastrongyloides trichosuri*	*Pleosporaceae* sp.
*Nippostrongylus brasiliensis*	*Torulaspora delbrueckii*
*Heligmosomoides polygyrus*	*Sarcoleotia turficola*
*Trichostrongylus colubriformis*	*Pneumocystis murina*
*Ancylostoma caninum*	**Amphibians**
*Dracunculus medinensis*	*Xenopus laevis*
*Dirofilaria immitis*	*Xenopus borealis*
	*Scinax rubra*
	*Phyllomedusa bicolor*
	*Rana chensinensis*
	*Bufo margaritifer*
	*Discoglossus pictus*

Nematodes selected represent a wide range of parasitic families, whilst fungi selected are known to have 18S sequences that commonly cross-react with nematode primers.

### PCR amplification

PCRs were prepared in aseptic conditions with all consumables UV sterilised, mastermixes were made on ice. PCRs were typically 25 μl in volume comprising: 10.88 μl of Mili-Q water, 2.5 mM PCR buffer, 3.5 mM Mg, 0.5 μM dNTPs, 0.024 U/μl FastStart Taq DNA Polymerase (Roche, Sussex, UK), 0.5 μM of both forward and reverse primers and 0.5 μl BSA (100X) (New England Biolabs Inc., Hitchin UK). 1 μl of tissue DNA extract was used, whilst between 5 and 10 μl of faecal DNA was used per reaction. Tissue DNA extracts typical contained 10–50 ng/μl and faecal extract from 4–63 ng/μl. Negative controls containing 5 μl of Milli-Q water instead of faecal or tissue DNA was run alongside PCRs to check for contamination. All primers were synthesised by Eurofins Genomics (Wolverhampton, UK). The *T*. *muris* specific primers were reported from Cutillas et al. (2002) whilst the nematode universal primers that were tested from the literature were from Bhadury and Austen (2010) and Floyd et al. (2005). The degenerate nematode specific primers developed in this study (Nem27 primers) comprised Nem1217F which had the 3’-5’ sequence CGN BCC GRA CAC YGT RAG and Nem1619 which had the 3’-5’ sequence GGA AAY AAT TDC AAT TCC CKR TCC. Nem27 primers amplify a 402 bp fragment of the 18S rRNA gene. DNA amplification was carried out using an initial denaturation at 94°C for 5 min; 35 cycles of amplification (94°C for 30 s; 54°C for 30 s; 72°C for 1 min); followed by a final extension at 72°C for 10 min. Nem27 primers could amplify nematode DNA from a faecal background at annealing temperatures as high as 62°C to 64°C, reducing the likelihood of non-specific amplification. All PCR amplifications were carried out in a Techne Prime Thermal Cycler (Staffordshire, UK) with a HYBAID touchdown compression pad (ThermoFisher). PCR product was kept chilled at 4°C.

### Gel electrophoresis

PCR products were run and visualised on 1% agarose gels comprising molecular grade agarose (Bioline, London, UK), TBE buffer and 0.5–2 μl GelGreen^TM^ Nucleic Acid Gel Stain (Biotium, Cambridge, UK). To load gel, 3 μl of PCR product was added to 2 μl of blue loading buffer (Bioline) and pipetted into the wells alongside 1 μl Hyperladder 1kb (Bioline) size standard. Product sizes were separated using electrophoresis in a RunOne^TM^ Electrophoresis Cell (Cheshire, UK) at 45 v for between 30–80 min, depending on the size of the gel. After separation, gels were drained, left to cool and then mounted on a PrepOne^TM^ Sapphire illuminator (EmbiTec) covered by a PI-1002 PrepOne^TM^ filter (EmbiTec) and camera hood and photographed.

### PCR product clean-up and Sanger sequencing

PCR product amplicons were cleaned using a MiniElute® PCR Purification Kit (Qiagen), with slight modifications to the manufacturer’s protocol. Cleaned DNA was eluted in 10 μl of autoclaved Milli-Q water for 20 minutes. 10–40 ng/μl of cleaned PCR product was added to 4 pmoles of a single relevant primer and the final volume adjusted to 10 μl using Milli-Q water. For each PCR amplicon one sample containing the forward and one the reverse primer was sent for sequencing. Samples were Sanger sequenced at the University of Manchester DNA Sequencing Facility using Big Dye 3.1 chemistry on an ABI 3100 Genetic Analyzer (Fisher Scientific).

### Sequence analysis

Sequence traces were examined and regions of poor quality or low-confidence sequence were removed in BioEdit. The complimentary sequence of that produced by the reverse primer was aligned next to the sequence produced by the forward primer, using the ClustalW function. This allowed for the extraction of the entire DNA sequence amplified by the primers. To identify the species from which the sequences were from they were run through the GenBank nucleotide BLAST tool (https://blast.ncbi.nlm.nih.gov/Blast.cgi?PAGE_TYPE=BlastSearch) and the top matches noted. Top matches always returned high query cover (99–100%) and maximum identity values (97–100%). Sequences reported in this study have been submitted to GenBank and their accession numbers are from MF535344 to MF535352.

### Faecal smears

Faecal pellets from *M*. *betsileo* amphibians were mounted on a glass slide with a few drops of Milli-Q water. The pellets were crushed and smeared over the slide, covered with a cover slip and sealed. Slides were then examined and photographed by light microscopy on a Leica S8APO Microscope at x80 magnification with a Leica MC 170HD video camera (Milton Keynes, UK).

## Results

### Development of a faecal DNA extraction protocol

**Fig 1:**
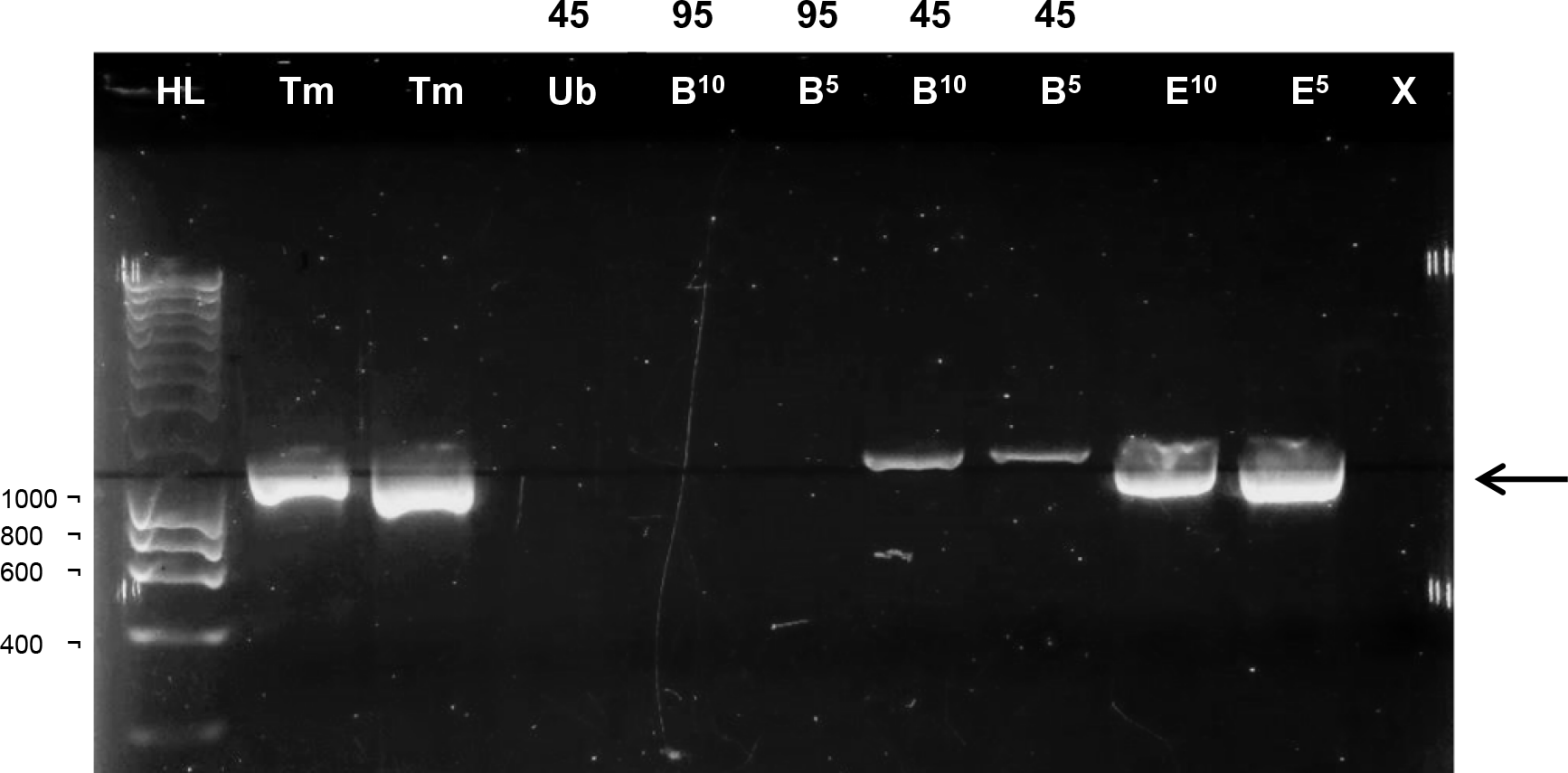
PCR amplification using *T*. *muris* primers on tissue, egg and faecal DNA. *T*. *muris* primers amplified DNA from *T*. *muris* tissue DNA (Tm) and *T*. *muris* eggs (E) beaten for 5 and 10 minutes (numbers in superscript). Faecal DNA from *T*. *muris* infected mice when unbeaten (Ub) did not amplify, as did faecal DNA that was beaten (B) but carried out at the DNA extraction lysis temperature of 95°C (Numbers in black above lanes in °C). Bead-beaten faecal samples amplified when the extraction lysis temperature was dropped to 45°C. Arrow indicates the position of the expected 1,000 bp product. 1kb hyperladders were run (HL) and negative controls (X).

To develop the faecal DNA extraction protocol a QIAamp® DNA stool mini kit was used on faeces from mice infected with *T*. *muris* nematodes to see if an eDNA signal could be detected, using nematode species specific primers from the literature [33,34]. When the manufacturer’s protocol was followed there was no successful amplification from faecal extracted DNA. Hence, to liberate parasite DNA from resilient transmissible stages a disruption step was added. A *T*. *muris* model of infection was used as eggs from this species are extremely tough and difficult to lyse [35]. The addition of a lysis step that used either 5 or 10 minutes of bead-beating permitted faecal eDNA amplification from mice infected with *T*. *muris* (Fig 1). Amplification did not occur at high lysis temperatures of 95°C but was possible when 45°C temperatures were used (Fig 1).

### Testing of designed primers and confirmation of specificity

Of the 28 primer pairs tested only eight amplified all nematode tissue DNA extracts (*T*. *muris*, *T*. *spiralis*, *A*. *lumbricoides* and *H*. *polygyrus*) and of these eight only two did not cross-react on faecal DNA from non-infected mice and tissue DNA from Platyhelminthes (*Schistosoma mansoni* and *Hymenolepis microstoma*). Cross-reactivity against Platyhelminth DNA was tested as other nematode specific primers from the literature [19,31] had previously been demonstrated to amplify DNA from this group, S1 and S2 Figs.

Testing of primers on faecal DNA from laboratory mice known to have no parasite infection acted as a negative control, ensuring a lack of primer cross-reactivity to DNA from other organisms found in faeces.

Of the two primer pairs that demonstrated no cross-reactivity, only one primer pair (Nem27 primers) amplified faecal DNA from mice infected with *T*. *muris* and *T*. *spiralis*. Nem27 primers also successfully amplified faecal DNA from captive colonies of the amphibians; *Mantella betsileo*, *M*. *aurantiaca*, *M*. *ebenaui*, *Dendrobates auratus* and *Agalychnis callidryas*, indicating infections.

Testing using annealing temperature thermal gradients found that Nem27 primers still amplified nematode eDNA from faeces at annealing temperatures as high as 62°C to 64°C. This produced tighter banding and reduces the possibility of primer cross-reactivity on DNA from outside of the Nematoda phylum, a factor which is particularly important given the Nem27 primers degeneracy and therefore increased potential to bind to non-target DNA.

**Fig 2:**
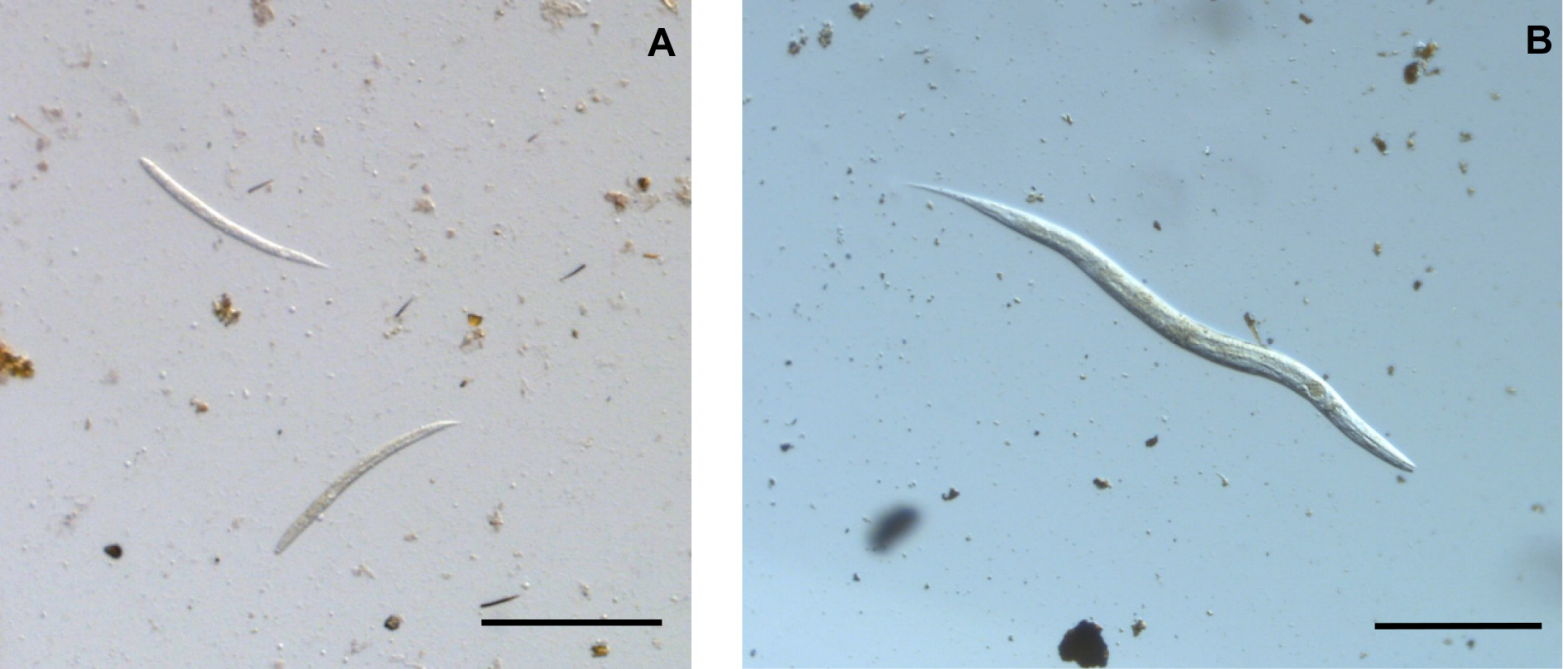
Light microscopy of *M*. *betsileo* faecal smears. Faecal smears from *M*. *betsileo* individuals were examined by light microscopy at x80 magnification. Nematode worm larvae (A) and adults (B) were observed. Bars are 100 μm.

Primer specificity was confirmed by sequencing, revealing that the Nem27 primers were binding at the expected region of the *T*. *muris* 18S rRNA gene. BLAST matches in GenBank returned a top match of *T*. *muris* when using the amplicon from the infected mouse faecal DNA and a top match from the genus *Poikilolaimus* from the *M*. *betsileo* faecal DNA. This data was supported by investigating faecal smears from *M*. *betsileo* by microscopy (Fig 2) which showed the presence of nematode worms.

### Applications of copro-diagnostic protocol with Nem27 primers to wild amphibians and captive herpetofauna

**Fig 3:**
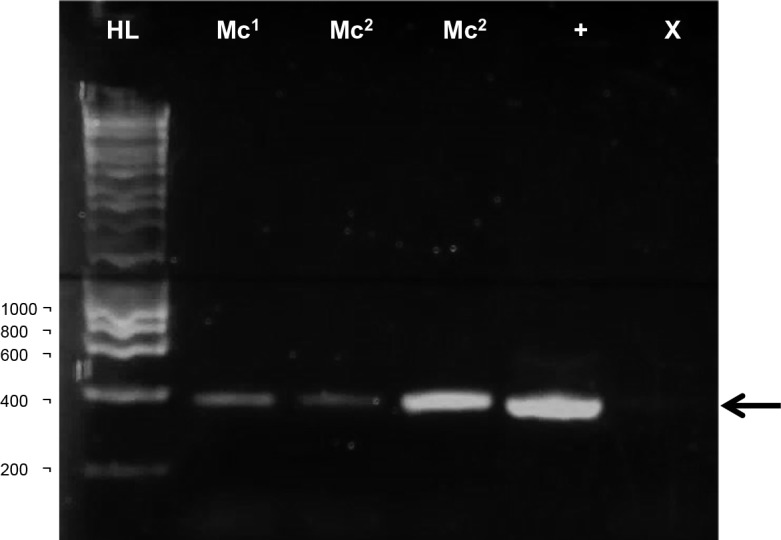
PCR amplification using Nem27 primers on faecal DNA from wild *M*. *cowani* amphibians. DNA was successfully amplified using the Nem27 primers on bead-beaten *M*. *cowani* faecal DNA, regardless of whether 1 or 5 minutes of bead-beating were employed. However, amplification was better when 5 minutes of bead-beating were used (Δ indicates 5 minutes of bead-beating). Both *M*. *cowani* faecal DNA extracts from different individuals amplified (numbers in superscript). A 40 cycle thermocycling program was chosen due to the low DNA concentrations obtained by the extraction (4.3 ng/μl) and permitted amplification. Such results indicate that these amphibians have nematode stages in their faeces and may therefore be infected. Arrow indicates the expected 402 bp size product. A positive control (+) containing 1 μl of tissue extracted *T*. *muris* DNA and 4μl of faecal DNA was included. 1kb hyperladder was run (HL) and a negative control (X).

Faecal samples from wild *M*. *cowani* that had undergone a 5 minute bead-beating step amplified better than those bead-beaten for one minute as indicated by a brighter band on the gel (Fig 3). This extraction obtained the lowest faecal DNA concentration of all extractions carried out in the present study (4.3 ng/μl), making 21.5 ng the known lower limit of total faecal DNA that Nem27 primers were able to amplify from. Amplicons produced were sequenced and returned a top match in GenBank from a nematode of the genus *Railletnema*. This genus lies phylogenetically within the Cosmocercidae, including species known to infect amphibians [36,37]. The next highest matches were from *Rhigonema ingens* and species of the genus *Heth* which are parasites of arthropods [38,39]. The fifth match was from the nematode parasite *Pseudonymus islamabadi* documented from the lizard, *Iguana iguana* [40].

**Fig 4:**
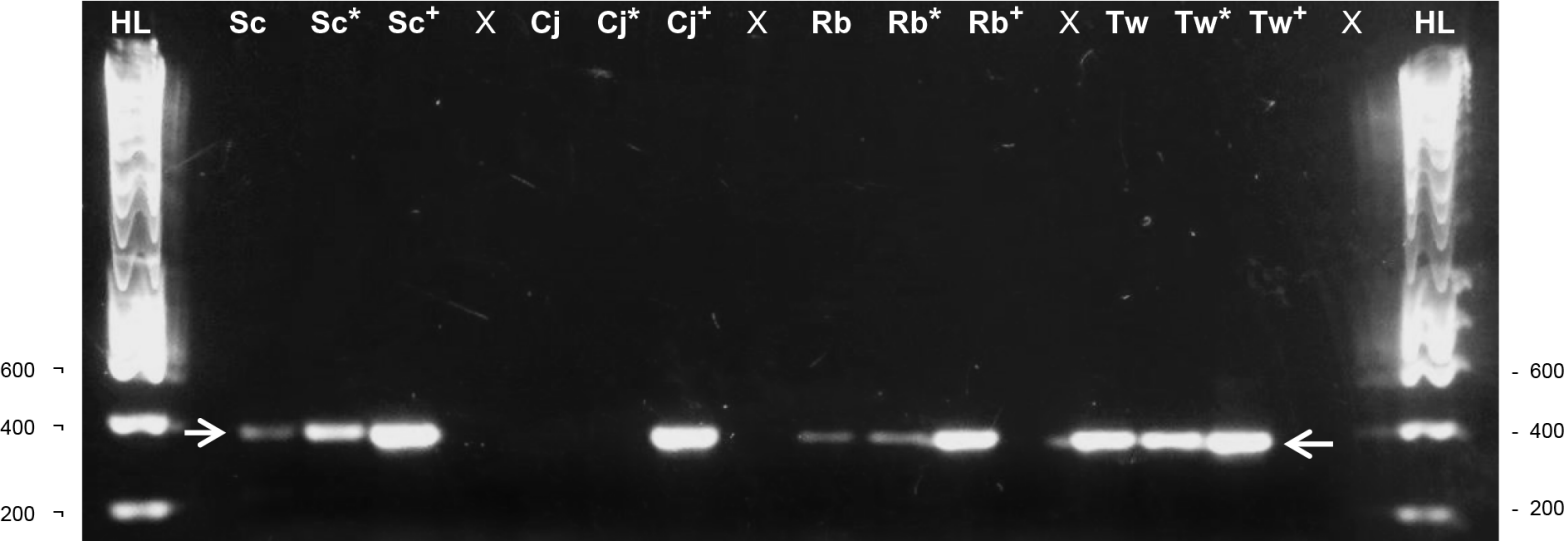
PCR amplification using Nem27 primers on faecal DNA from ZSL London Zoo reptiles. Nem27 primers successfully amplified both 5 μl and 10 μl (asterisked) of faecal DNA from *S*. *crocodilurus* (Sc), *R*. *boulengeri* (Rb), and *T*. *g*. *whitei* (Tw) indicating a likely nematode infection in these reptile species but not from *Chamaeleo jacksoni* (Cj) which exhibited no amplification. Arrows indicate the expected 400 bp size product. Positive controls (+) containing 1 μl of tissue extracted *T*. *muris* DNA and 4 μl of the relevant reptile faecal DNA were included, demonstrating an absence of PCR inhibitors in these extracts. 1kb hyperladders were run (HL) and negative controls (X).

30 faecal samples from 7 different amphibian and 17 different reptile species maintained at ZSL London Zoo were also analysed. Six samples yielded amplification products when either 5 μl or 10 μl of faecal DNA was used. The following herpetofauna species produced an amplification signal: *Phyllobates bicolor*, *Dendrobates tinctorius*, *Shinisaurus crocodilurus*, *Rhynchophis boulengeri*, *Testudo graeca floweri* and *T*. *g*. *whitei*. These results indicate the presence of nematode eDNA in these faecal DNA extracts and therefore a possible parasitic nematode infection. An example of successful amplification from three reptile species is shown (Fig 4).

Sequencing of amplicons from the *D*. *tinctorius*, *S*. *crocodilurus*, *T*. *g*. *whitei* and *T*. *g*. *floweri* hosts returned top matches from nematode species and genera known to be parasitic. The top match for the two tortoise species, *T*. *g*. *whitei* and *T*. *g*. *floweri*, was from the pinworm species *Aspiculuris tetraptera* which infects laboratory mice, alongside other vertebrates [41,42]. The next match, *Ozolaimus linstowi* is known to be a parasite of lizards [40]. The top nematode sequence match for the amphibian host *D*. *tinctorius*, was from the *Railletnema* genus the same as that found in the *M*. *cowani* hosts. The sample from the host lizard, *S*. *crocodilurus*, obtained top matches with the nematode genus *Diploscapter* a genus that contains both parasitic and free-living species [43,44].

The sequenced amplicons from *P*. *bicolor* and *R*. *boulengeri* both returned top matches with *Oscheius tipulae* and *Poikilolaimus oxycercus* both recognised as common non-parasitic soil dwelling nematodes [45,46].

## Discussion

Declines in global biodiversity continue despite efforts to alleviate the situation, with many factors and synergies between anthropogenic effects and natural ecological processes as yet poorly understood [1,2,47]. Species losses in the amphibian class are possibly the most severe among terrestrial vertebrates, with many previously abundant species now extinct and numerous others still threatened [3,5]. Now, studies are beginning to shed light on the role metazoan parasites are playing in this crisis, weakening already susceptible populations in the wild or causing die-offs in *ex situ* colonies intended for species conservation [13,48,49]. Hence, effective techniques are needed for detecting parasitic infection that are non-damaging to host populations, unlike necropsy, or that are more sensitive than common non-invasive methods, e.g. microscopy on faecal smears [8,50]. Molecular based copro-diagnostic detection and barcoding of eDNA presents a viable alternative and has already been used in other hosts to successfully track and discover reservoirs of zoonotic parasite infections, such as ancylostomiasis [51], trichuriasis and echinostomiasis [52].

Here, we have developed an effective alternative and created a novel copro-diagnostic molecular technique capable of liberating and detecting eDNA shed in faeces from amphibian, as well as reptile and mammal, hosts. We have also designed a novel pair of nematode universal (Nem27) primers capable of binding to tightly conserved regions of the 18S rRNA gene from a variety of nematode species. Sequencing and comparison in GenBank of amplicons produced by these Nem27 primers demonstrated their specificity for nematode DNA. In addition, testing of Nem27 primers on faecal DNA from non-infected and infected mice (using *T*. *muris* and *T*. *spiralis* infection models) assisted in confirmation of their specificity to nematode DNA alone.

Key findings made included identification of infection in the Madagascan frog *M*. *cowani* by a nematode of the genus *Railletnema*, a genus known to contain at least 22 species of amphibian parasites [36,37]. *D*. *tinctorius* dart frogs from ZSL London Zoo were also infected with nematodes from this genus. This species has historically been diagnosed with ‘very numerous helminth larval forms’ in faecal smears but the identity of helminths had not previously been established (C. Michaels, pers. comm., October 15, 2016). In addition, our method highlighted a potential pinworm infection by *A*. *tetraptera* or close relative, in two tortoise species from samples provided by ZSL London Zoo. These results corroborated separate findings made by staff at ZSL London Zoo that had previously identified ‘Strongyle-like ova’ and ‘moderate *Tachygonetria* ova’ in the faeces of two tortoises (C. Michaels, pers. comm., August 25, 2016). Members of the genus *Tachygonetria* are near relatives of *A*. *tetraptera* and are also in the Oyxuridae family [53,54]. However, due to *A*. *tetraptera* being a common pinworm infection in rodents [55] there exists a small likelihood of enclosure contamination by frozen murine material used to feed other carnivorous reptile species. In all cases, potentially infected animals at ZSL London Zoo and the University of Manchester were clinically healthy animals and were not showing signs or symptoms of parasitoses. At ZSL London Zoo, faeces is routinely screened for elevated or pathological parasitoses and a strategy of management of normal parasite loads, rather than elimination of all gut metazoan, is implemented (C. Michaels, pers. comm., November 10, 2016). In fact, some parasitic infection is entirely expected, often in the complete absence of clinical signs of infection and is an important driver of individual immune competency and overarching ecological structure and function [56]. Thus, our copro-diagnostic technique could be applied to shed light on natural nematode biodiversity in both wild and captive amphibian host populations, accruing data that would alternatively require lengthy amounts of microscopic examination.

A number of unexpected caveats within the copro-diagnostic method were also revealed, including the detection of eDNA from common free-living bacterivorous nematodes. Given the ubiquitous nature of nematodes it is unsurprising that individuals may have migrated from the soil compartment of the terraria into the amphibian faeces. Studies investigating the effects of organic soil amendments have found that addition of manure to soils causes distinct increases in the number of bacterivorous free-living nematodes present [57]. This is thought to arise due to manure increasing the bacterial content of the soil, followed by heightened predation and proliferation by bacterivorous nematodes [58]. Moreover, the common bacterivore, *Caenorhabditis elegans* has been observed to display preferences for different manure types, migrating into faeces following trails of faecal compounds released into the soil [59]. Thus, the issue of detection of DNA from free-living nematodes in faeces may reflect the fact that many nematodes exhibit a preference for the faecal microhabitat.

Contamination by non-parasitic nematodes is relatively unique to the current study, owing to the fact that the molecular detection system was developed to detect all nematodes given the dearth of information regarding the common parasites of amphibians. Parasitism has evolved independently in the Nematoda phylum many times in different clades, making the identification of targetable, conserved DNA sequences in parasitic groups that are absent in non-parasitic ones unlikely [60]. However, sequencing of amplicons produced can quickly identify which positive results are from true infections. Furthermore, PCR tends to amplify the more abundant sequences in a DNA extract [61]. Hence, in faecal samples from a heavily infected host the eDNA signal in the faeces is likely to be stronger and outcompete any potential contaminant DNA from free-living nematodes [61].

Our study also highlighted some potential difficulties with using the 18S rRNA gene for effective nematode barcoding. Sequenced amplicons generated using the Nem27 primers frequently returned high matches with existing sequences in the GenBank database; however, these were often from nematodes of differing families and genera, providing poor consensus as to the exact species present. For example, the top nematode matches from the ratsnake, *R*. *boulengeri* were predominantly from the free-living genus *Poikilolaimus* [46]. Nonetheless, the fourth match which had an equivalent query cover and sequence identity belonged to the genus, *Krefftascaris* known to be common parasites of turtles [62]. In this case, the *R*. *boulengeri* snakes had repeatedly shown no signs of nematode infection when tested using traditional faecal screening, suggesting that environmental contamination with *Poikilolaimus* is more likely than infection with *Krefftascaris* (C. Michaels, pers. comm., October 15, 2016).

In addition, matches obtained in GenBank frequently returned sequences annotated as ‘Uncultured Eukaryote clone’, providing no data on the identity of the matching sequence and therefore no help in identification of the query sequence. A number of studies have found fault with the quality of sequence metadata in GenBank, highlighting the prevalence of absent or poor taxonomic resolution provided with sequences, alongside a lack of country of origin and ecological data [63,64]. Furthermore, even if a taxonomic identification based on morphology is provided there is no way of guaranteeing its accuracy [63,65]. Other, more regulated databases could be used in future studies, such as that maintained by The International Barcode of Life (iBOL) project [63,66]. This project’s database uses a 650 bp region of the COI gene to barcode all animal life and is compiled of standardised DNA sequences that have come from museum and voucher specimens with thorough taxonomic identification [63,66]. The quality of such data is rigorously checked, permitting effective comparison of sequences between species and clades for more accurate phylogenetic investigation [63].

In summary, we have realised a novel molecular methodology, demonstrating that eDNA released from parasitic nematodes can be detected in the faeces of amphibian, reptile and mammalian hosts and therefore provide important information on these organisms infection status. With some refinement, to be truly independent of post-mortem examination of hosts, our protocol lays down a crucial framework upon which further development may potentiate its use for the conservation of ecologically significant bioindicator groups, such as the amphibians [67]. Future work may explore the potential of using the Nem27 primers developed here in a real-time PCR format to provide quantitative data on parasite eDNA in host faeces and therefore provide a potential proxy for parasite burden [24]. Such modifications could give, our protocol utility as a quantitative diagnostic in the veterinary sciences where wild parasites may be infecting livestock, or to advance general scientific understanding of wild host-parasite systems, providing information on the dynamics of parasite populations [24,68].

## Supporting information

S1 Table ZSL, London Zoo herpetofauna species faecal samples. (PDF)

S1 Fig Test for published^[^^31^^]^ nematode universal primer efficacy on nematode tissue DNA and cross-reactivity on Platyhelminth tissue DNA. A: Nematode universal primers [31] designed for specific amplification of nematode DNA successfully amplified DNA from the nematodes *T*. *muris* (Tm) and *T*. *spiralis* (Ts). B, C: Nematode universal primers [31] also demonstrated cross-reactivity on *S*. *mansoni* (Sm) and *H*. *microstoma* (Hm) tissue DNA producing multiple bands, including a strong band at the expected 900 bp (arrows). Numbers in superscript indicates whether the PCR was carried out at an annealing temperature of 59.4°C (1) or 60.3°C (2). 1kb hyperladders were run (HL) and negative controls (X).
(TIF)

S2 Fig Test for published^[^^19^^]^ nematode universal primer efficacy on nematode tissue DNA and cross-reactivity on Platyhelminth tissue DNA. Nematode universal primers [19] designed for specific amplification of nematode DNA successfully amplified DNA from the nematodes *T*. *spiralis* (Ts), *A*. *lumbricoides* (Al), *N*. *brasiliensis* (Nb), *H*. *polygyrus* (Hp) but not *T*. *muris* (Tm). These primers also demonstrated cross-reactivity on *S*. *mansoni* (Sm) and *H*. *microstoma* (Hm) tissue DNA. Arrow indicates the expected 427 bp size product. 1kb hyperladders were run (HL) and negative controls (X).
(TIF)
