# Correction: A novel copro-diagnostic molecular method for qualitative detection and identification of parasitic nematodes in amphibians and reptiles

**DOI:** 10.1371/journal.pone.0198977

**Published:** 2018-06-07

**Authors:** Lucas G. Huggins, Christopher J. Michaels, Sheena M. Cruickshank, Richard F. Preziosi, Kathryn J. Else

There is an error in the eighth sentence of the PCR amplification section. The correct sentence is: The degenerate nematode specific primers developed in this study (Nem27 primers) comprised Nem1217F which had the 5’-3’ sequence CGN BCC GRA CAC YGT RAG and Nem1619 which had the 5’-3’ sequence GGA AAY AAT TDC AAT TCC CKR TCC.
