# Supplementary material for: A novel copro-diagnostic molecular method for qualitative detection and identification of parasitic nematodes in amphibians and reptiles
Source: PLoS One. 2017 Sep 21;12(9):e0185151. doi: 10.1371/journal.pone.0185151 (PMC5608329; doi:10.1371/journal.pone.0185151)
Supplement: S1 Table — (PDF) [file pone.0185151.s001.pdf]

**Amphibia**	**Collection Date**	**ZIMS ID**
Alytes muletensis	11.06.16	9432-9442
Alytes obstetricans	11.06.16	G00064
Ambystoma dumerilii	11.06.16	G02030
Dendrobates tinctorius	20.06.16	N/A
Leptodactylus fallax	14.06.16	UMC Pen 3
Neurergus kaiseri	20.06.16	N/A
Phyllobates bicolor	11.06.16	G01026
**Reptilia**	**Collection Date**	**ZIMS ID**
Abronia graminea	12.06.16	G01454-G01455
Aspidites melanocephalus	19.06.16	G01329
Chamaeleo jacksonii	11.06.16	E0163
Ctenosaura bakeri	09.06.16	3064
Egernia stokesii	09.06.16	C01922
Gekko gecko	17.06.16	G02069
Gekko gecko	11.06.16	3251-3252
Gekko gecko	17.06.16	4188
Gekko gecko	11.06.16	G0270
Heloderma horridum	19.06.16	3858
Hydrodynastes gigas	20.06.16	5082
Pelodryas caerulea	11.06.16	G01013-G01024
Pelodryas caerulea	09.06.16	N/A
Plica plica	12.06.16	G01011-G01012
Rhynchophis boulengeri	12.06.16	G01177
Shinisaurus crocodilurus	17.06.16	G01840-G01843
Shinisaurus crocodilurus	19.06.16	G01842
Simalia boeleni	13.06.16	9396
Simalia boeleni	11.06.16	9398
Simalia boeleni	12.06.16	7076-7077
Testudo graeca floweri	09.06.16	C779
Testudo graeca whitei	09.06.16	C707
Varanus prasinus	11.06.16	7842
