# Supplementary figures and images for: A novel copro-diagnostic molecular method for qualitative detection and identification of parasitic nematodes in amphibians and reptiles
Source: PLoS One. 2017 Sep 21;12(9):e0185151. doi: 10.1371/journal.pone.0185151 (PMC5608329; doi:10.1371/journal.pone.0185151)

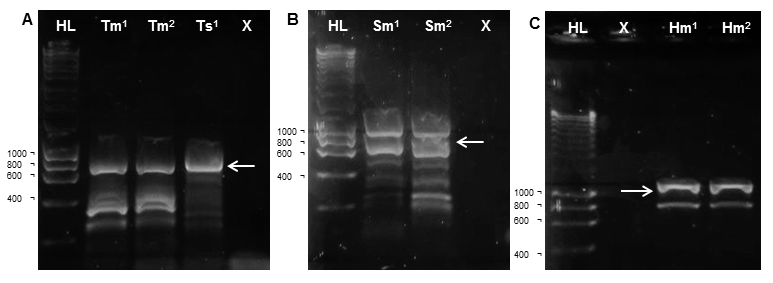

Supplement: S1 Fig — A: Nematode universal primers [31] designed for specific amplification of nematode DNA successfully amplified DNA from the nematodes T. muris (Tm) and T. spiralis (Ts). B, C: Nematode universal primers [31] also demonstrated cross-reactivity on S. mansoni (Sm) and H. microstoma (Hm) tissue DNA producing multiple bands, including a strong band at the expected 900 bp (arrows). Numbers in superscript indicates whether the PCR was carried out at an annealing temperature of 59.4°C (1) or 60.3°C (2). 1kb hyperladders were run (HL) and negative controls (X). (TIF) [file pone.0185151.s002.tif]

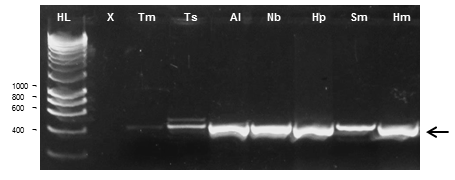

Supplement: S2 Fig — Nematode universal primers [19] designed for specific amplification of nematode DNA successfully amplified DNA from the nematodes T. spiralis (Ts), A. lumbricoides (Al), N. brasiliensis (Nb), H. polygyrus (Hp) but not T. muris (Tm). These primers also demonstrated cross-reactivity on S. mansoni (Sm) and H. microstoma (Hm) tissue DNA. Arrow indicates the expected 427 bp size product. 1kb hyperladders were run (HL) and negative controls (X). (TIF) [file pone.0185151.s003.tif]
